# Repetitive DNA is Functional and Encodes Parts of the Non‐Coding RNA Repertoire

**DOI:** 10.1002/ggn2.202200026

**Published:** 2022-11-26

**Authors:** James A. Shapiro

**Affiliations:** ^1^ Department of Biochemistry and Molecular Biology University of Chicago Chicago IL 60637 USA

## Abstract

This is a commentary on the article by Eviatar Nevo and Kexin Li entitled “Sympatric Speciation in Mole Rats and Wild Barley and Their Genome Repeatome Evolution: A Commentary”, published recently in *Advanced Genetics*.

Eviatar Nevo and Kevin Li just published a paper analyzing the genome sequences of sympatric species of subterranean mole rats (*Spalax*) and wild barley (*Hordeum*) from the Evolution Canyon and other sites in Israel where short distances separate habitats with very different ecologies.^[^ ^1^ ^]^ The ecological differences drive habitat‐specific adaptations and sympatric speciation events.

What makes Nevo and Li's paper particularly interesting is that the authors analyzed separately the protein‐coding and noncoding (largely repetitive) genome sequence relationships in drawing molecular phylogenies of these mole rat and barley populations, determining branch lengths by the abundance of single nucleotide polymorphisms (SNPs). Most evolutionists accept the proposition that the relatedness and descent patterns of coding sequences will reflect the selective effects and constraints imposed by ecological divergences on the evolving species genomes. However, those who believe that such selective effects and constraints do not apply to noncoding DNA, often referred to as “selfish DNA” because of its repetitive content,^[^ ^2^ ^]^ would expect a different pattern of sequence relationships, determined more by events like bursts of repeat sequence amplifications than ecological divergences. Thus, it is all the more striking that Nevo and Li find that species phylogenies determined by noncoding DNA exactly mirror (i.e., match) those from coding sequence DNA, both for the mole rat and for barley, as illustrated here (**Figure** **1**).

**Figure 1:**
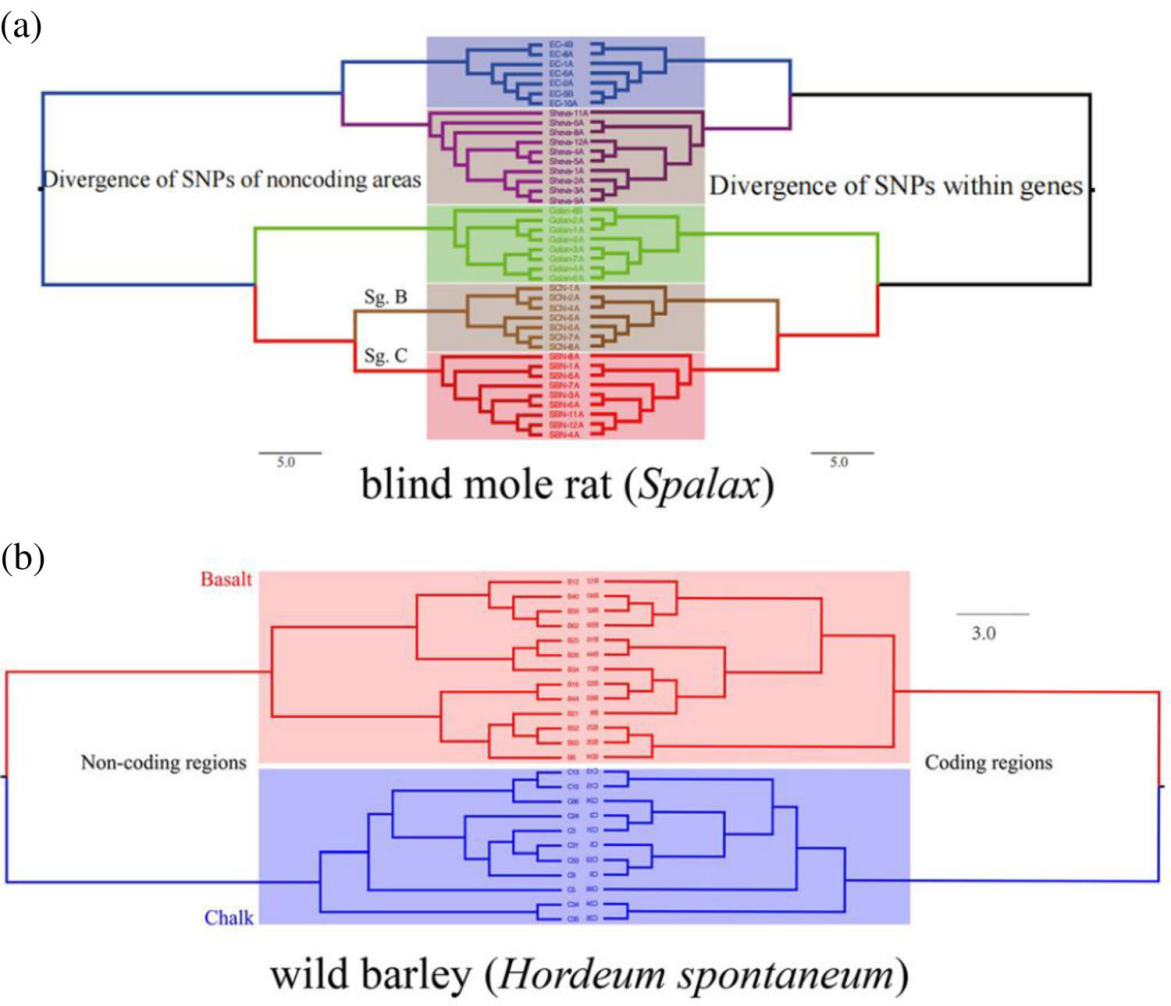
This is taken from Figures 11 and 12 of Nevo and Li.^[^ ^1^ ^]^ Neighbor‐joining phylogenetic trees linking sympatric species of (a) blind mole rat (*S. galili*) and (b) wild barley (*Hordeum spontaneum*), from the ecologically diverse “Evolution Canyon” and “Evolution Plateau”, Upper Galilee Israel, based on the SNPs from coding and noncoding genomic regions. The numbers in the middle indicate the genotypes studied in each population. Note, the mirror image of the coding and non‐coding genomes demonstrating that both are subjected to the same ecological stresses and are selected accordingly. Therefore, the non‐coding genome cannot be either “Junk” or “selfish” DNA” but appears to be regulatory as in mole rat Spalax. Reproduced under the terms of the CC‐BY license.^[^ **^1^** ^]^ Copyright 2022, the Authors. Published by Wiley Periodicals LLC.

What do these striking congruences mean? Nevo and Li state that these results indicate that noncoding sequences are subject to the same ecological pressures as coding sequences, which they attribute to the *cis*‐regulatory functions of repetitive DNA sequences. While it is correct that genomic recognition sequences for polymerases, transcription factors, architectural proteins, and other molecules that interact with the genome in a sequence‐dependent manner will be subject to ecological constraints as much as protein‐coding sequences, there is another dimension to the functional significance of repetitive and other so‐called “noncoding DNA.”

The repetitive elements in the genome actually do encode functional molecules that happen not to be proteins but are instead many classes of non(protein)coding ncRNAs. These ncRNAs execute a rapidly growing list of important tasks in the lives of complex organisms like mammals and grains, as we can see from the following list of diverse characters affected by ncRNA loss:

MicroRNAs (miRNAs): Plant miRNAs are key gene expression regulators that control development, defense against invading pathogens, and adaptation to abiotic stress;^[^ ^3^ ^]^ in *Drosophila*, miRNAs contribute to tissue growth, germ cell development, hormone action, and the development and activity of the central nervous system as well as organismal robustness;^[^ ^4^ ^]^ miRNAs control the abundance of the majority of the vertebrate transcriptome^[^ ^5^ ^]^ and also coordinate stress responses and facilitate apoptosis in animals.^[^ ^6^, ^7^ ^]^

Small interfering RNAs (siRNAs) carry out translation repression and stress adaptation in plants,^[^ ^8^ ^]^ are important in chromatin maintenance in *Arabidopsis*,^[^ ^9^ ^]^ in *Drosophila* embryonic development,^[^ ^10^ ^]^ in germline development in higher metazoa,^[^ ^11^ ^]^ and for maintaining longevity in nematodes.^[^ ^12^ ^]^

Piwi‐interacting RNAs (piRNAs) were first identified as silencers of mobile genetic elements in the *Drosophila* germline stress response.^[^ ^13^ ^]^ *Drosophila* piRNAs also influence embryonic development,^[^ ^14^ ^]^ and a somatic piRNA controls fat body metabolism.^[^ ^15^ ^]^ All metazoa have piRNAs for a wide range of similar tasks.

Long noncoding RNAs (lncRNAs) regulate genome organization and expression by bringing together distal chromosomal regions (e.g., enhancers and promoters),^[^ ^16^, ^17^ ^]^ by splicing control,^[^ ^18^ ^]^ and also facilitate cellular metabolism by serving as scaffolds for multi‐component complexes and phase‐separated liquid condensates;^[^ ^19^ ^]^ in addition, lncRNAs influence many specialized functions like DNA damage response,^[^ ^20^ ^]^ interferon response,^[^ ^21^ ^]^ epidermal–mesenchymal transition (EMT) in cancer,^[^ ^22^ ^]^ and X inactivation in female cells.^[^ ^23^, ^24^ ^]^

This is not to say that all functional ncRNAs are templated exclusively by repetitive DNA. Nonetheless, many of the shorter miRNAs, siRNAs, and piRNAs do contain only repetitive sequences.^[^ ^25^, ^26^, ^27^ ^]^ In addition, many lncRNAs contain significant regions that originated from repetitive and mobile DNA elements,^[^ ^28^, ^29^ ^]^ and those regions may be critical to their functionalities.^[^ ^30^ ^]^

In other words, our knowledge of genome coding has expanded tremendously since the 20th century, when the genome was considered basically a database of protein sequences. Today, we realize that formerly ignored repetitive genome components template the transcription of important biomolecules and participate actively in determining cellular and organismal characters. The whole genome contributes to the organism and, as Nevo and Li show us, evolves responding to ecological inputs in sympatric speciation as a single integrated entity.

## Conflict of Interest

The author declares no conflict of interest.
